# Supplementary material for: Spectral-temporal-spatial customization via modulating multimodal nonlinear pulse propagation
Source: Nat Commun. 2024 Mar 6;15:2031. doi: 10.1038/s41467-024-46244-5 (PMC10918100; doi:10.1038/s41467-024-46244-5)
Supplement: Supplementary file 1 — Supplementary Information [file 41467_2024_46244_MOESM1_ESM.pdf]

Spectral-temporal-spatial customization via modulating multimodal nonlinear pulse propagation

Tong Qiu[1†], Honghao Cao[1†], Kunzan Liu[1], Li-Yu Yu[1], Manuel Levy[2], Eva Lendaro[2], Fan Wang[2], Sixian You[1*]

[1]Department of Electrical Engineering and Computer Science, Massachusetts Institute of Technology, Cambridge, MA, USA.
[2]Department of Brain and Cognitive Sciences, Massachusetts Institute of Technology, Cambridge, MA, USA.

*Corresponding author(s). E-mail(s): sixian@mit.edu;
†These authors contributed equally to this work.

Supplementary Note 1. Multimodal nonlinear pulse propagation and spectral broadening in the SI MMF

To gain insights into the broadening and the tuning mechanisms, we first investigated the evolution of the output spectra with gradually increasing input pulse energy by using the characterization setup as depicted in Fig. 2. The fiber shaper was set to its initial state with no bending applied to the fiber. The results are presented in Supplementary Fig. 1a. As the injected pulse energy gradually increases, SPM, XPM, and intermodal FWM begin to dominate the spectral broadening process. As the input energy increases further, the output spectrum extends to the anomalous dispersion regime and multimode solitons (i.e., solitary waves consisting of multiple spatial modes) begin to form, due to the balancing between the nonlinear phase modulation and dispersive effects including the intramodal group velocity dispersion and intermodal velocity mismatch [1]. Meanwhile, excess energy from the soliton fission process is emitted as dispersive waves through the interaction between the nonlinear and higher-order dispersion effects. The dispersive waves are generated at shorter wavelengths to satisfy the phase-matching condition [2]. With further increase in input energy, the multimode soliton undergoes a redshift, resembling soliton self-frequency shifting [3], and eventually the spectrum extends beyond the near-infrared-II region. Accordingly, the phase-matched dispersive waves shift towards the visible region. These observations are in agreement with the findings reported in [2, 4, 5] with input wavelengths deeper into the anomalous dispersion regime.

In addition, the fiber output power at 800-nJ input pulse energy was monitored for 1 hour to assess its stability (Supplementary Fig. 2), which was measured to reflect 1–2% fluctuations at different wavelengths. Despite exceeding the critical power for catastrophic self-focusing (about 8 MW for silica at 1300 nm [6]), the pronounced dispersion effects associated with ultrashort pulses can effectively modify the dynamics of self-focusing, thereby preventing the exponential increase in peak intensity [7]. In this study, the fiber source was stable enough to generate reproducible output modulation and biological images (see later sections). Nevertheless, the fluctuation metric could potentially be improved by using a better power meter (low dynamic range was used for the wavelength range above 1100 nm in this study), a more environmentally controlled optical space (temperature and humidity), and a more isolated fiber configuration (gel or air isolation).

To validate our experimental results, we performed numerical simulations (Supplementary Fig. 1b) using the simplified (1+1)D model based on the generalized multimode nonlinear Schrödinger equation (GMMNLSE) [8, 9] (see Methods) with the same fiber parameters and launching conditions as in the experiments. Only the first seven radially symmetric modes are included to save computation time. Overall, we observed a good agreement in the spectral broadening between the experimental and numerical results at all energy levels. The discrepancy in the distribution of power spectral density is likely due to the limited number of modes in the simulation, which may not fully capture the multimodal nonlinear broadening in experiments.

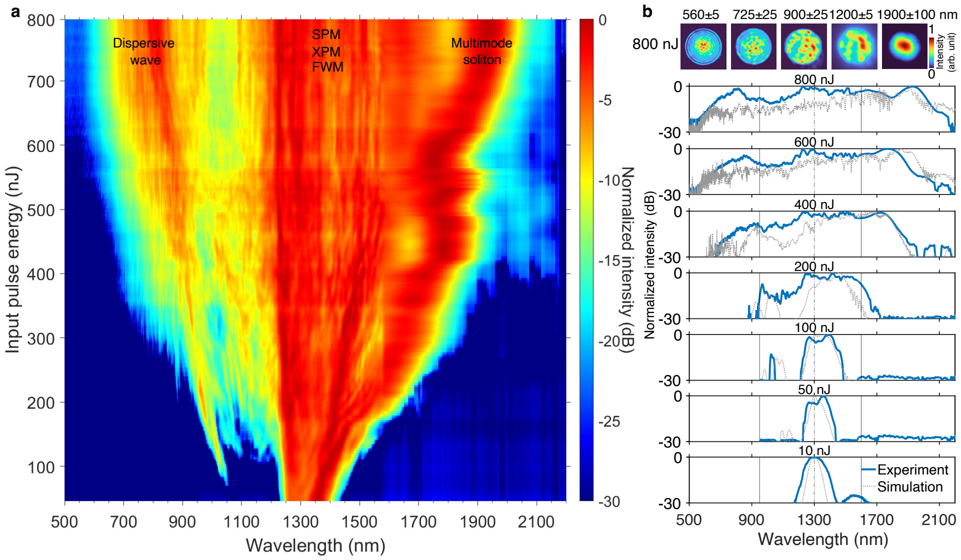

Supplementary Figure 1: **Multimodal spectral broadening in SI MMF. a**, Spectral evolution as a function of increasing input pulse energy in a 30-cm-long SI MMF, showing the emergence of three spectral windows dominated by distinct nonlinear effects. **b**, Output spectra at selected input pulse energies. The top row presents the spectrally filtered near-field beam profiles at an input energy of 800 nJ, numbers above each profile denote the center wavelength and bandwidth of the bandpass filter applied. Numerical simulations are presented in dotted gray, with the seven modes assumed to be excited with equal energy and phase. For visualization purposes, the spectrum and beam profile at each input pulse energy are normalized to their respective peak values at that energy. Source Data are provided as a Source Data file.

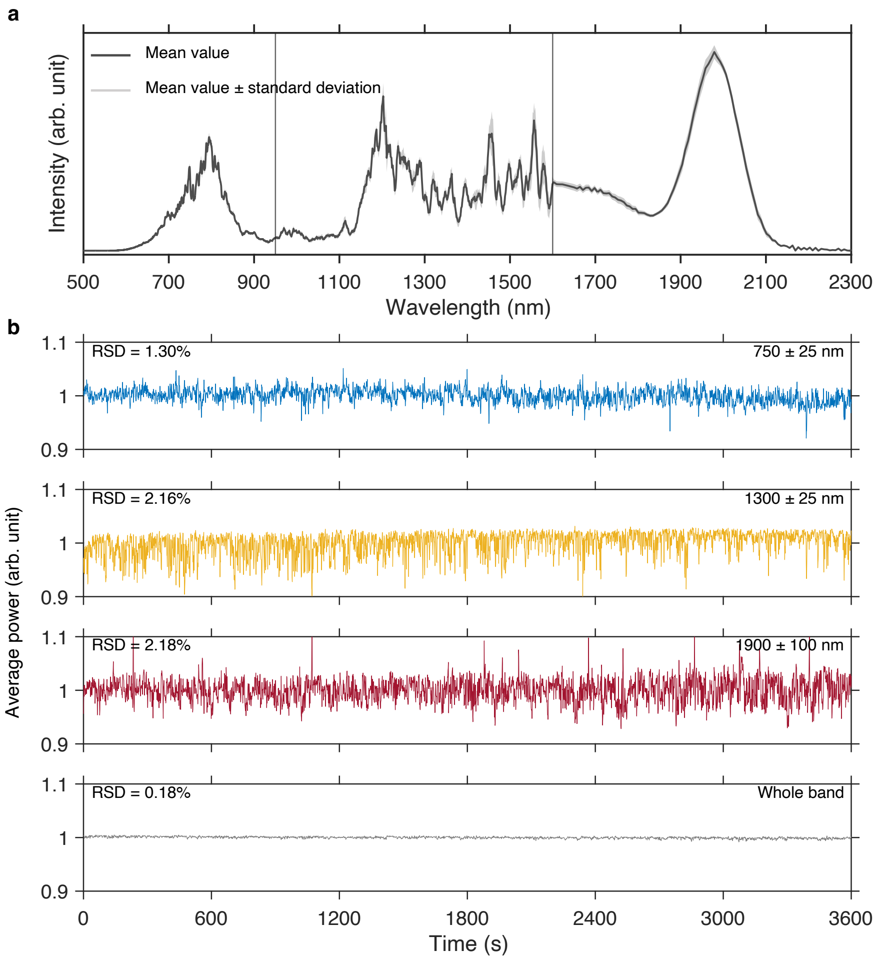

Supplementary Figure 2: **Stability of the MMF source.** **a**, Spectral stability of the SI MMF source obtained from the output spectra recorded per second for a period of 1 hour. The solid black trace represents the mean value, and the surrounding gray region indicates the level of variation (quantified by the standard deviation) of the recorded spectra. **b**, Normalized output average power of the SI MMF source recorded per second for a period of 1 hour. A silicon-based photodiode sensor (Thorlabs PM16-130) was used to measure the optical power at wavelength below 1100 nm and a thermal power sensor (Thorlabs S425C-L) was used to measure the optical power of longer wavelengths and the whole band. The SI MMF was pumped by 800-nJ 46-fs pulses at 1300 nm. RSD: relative standard deviation. Source data are provided as a Source Data file.

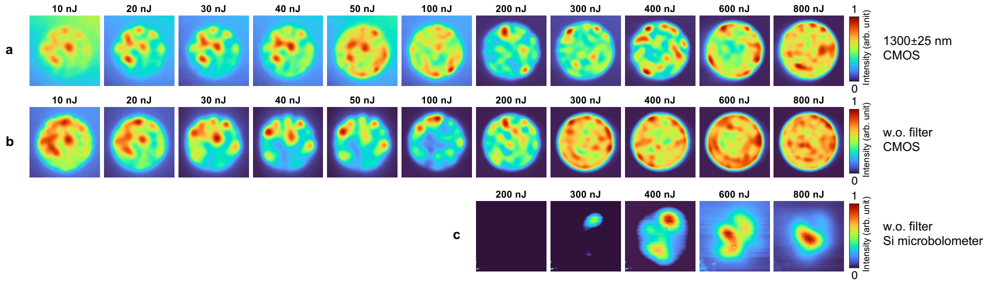

Supplementary Figure 3: **Multimodal spectral broadening in SI MMF**. **a**–**c**, Representative results of near-field beam profiles out of a 30-cm-long SI MMF at different input pulse energies (1300 nm input wavelength) acquired using a CMOS camera (Mako, G-040B) with (**a**) and without (**b**) a bandpass filter; and a thermal imaging camera based on silicon (Si) microbolometer (DataRay, WinCamD-FIR2-16-HR) with no bandpass filter applied (**c**).

Supplementary Note 2. Optimization of the fiber shaper device

The fiber shaper enables spatiotemporal control of the nonlinear effects in SI MMFs, underlying great tunability in the fiber source properties to adapt to diverse applications. To maximize this tunability, we investigate several key parameters involved in the design of the fiber shaper. These parameters include the number of actuators, as well as the total range and resolution of the actuator's linear motion. The impact of these parameters on the 900–1700 nm band are shown in Supplementary Figs. 4–6, where we define an average spectral tuning ratio (η) to quantify the tunability:

$$\eta = \frac{1}{N} \sum_{n=1}^{N} \frac{I_{\lambda_n}^{\max}}{I_{\lambda_n}^{\min}}. \tag{S1}$$

N denotes the number of discrete wavelengths acquired by the spectrometers, $I_{\lambda_n}^{\max}$ and $I_{\lambda_n}^{\min}$ refer to the maximum and minimum intensity at each wavelength. Our results indicate that the performance is optimized when using 5 actuators, a displacement range of 20 mm, and an actuator motion resolution of 5 mm. Notably, these parameters are specifically tailored to this device and may require adjustments for devices with different physical dimensions.

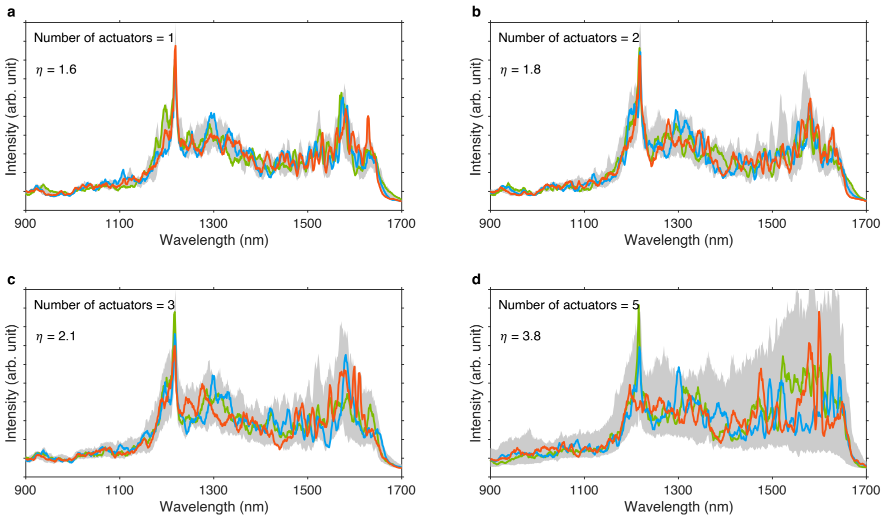

Supplementary Figure 4: **Spectral tunability of the fiber shaper with different numbers of actuators. a–d**, The output spectra range of the fiber-shaper-controlled SI MMF with 1 (**a**), 2 (**b**), 3 (**c**), and 5 (**d**) actuators, respectively. The input wavelength is set at 1300 nm. Source data are provided as a Source Data file.

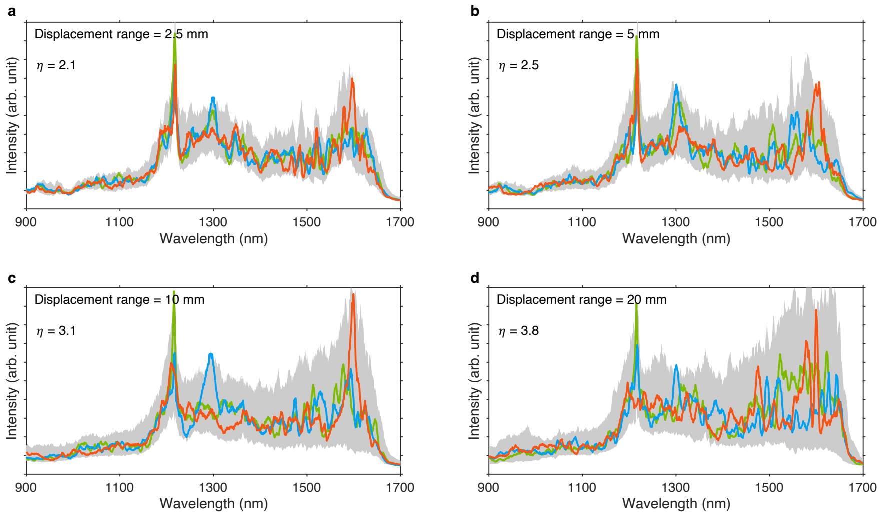

Supplementary Figure 5: **Spectral tunability of the fiber shaper with different displacement ranges. a–d**, The output spectra range of the fiber-shaper-controlled MMF with a displacement range of 2.5 mm (**a**), 5 mm (**b**), 10 mm (**c**), and 20 mm (**d**), respectively, all with 5 actuators and a motion resolution of 5 mm. The input wavelength is set at 1300 nm. Source data are provided as a Source Data file.

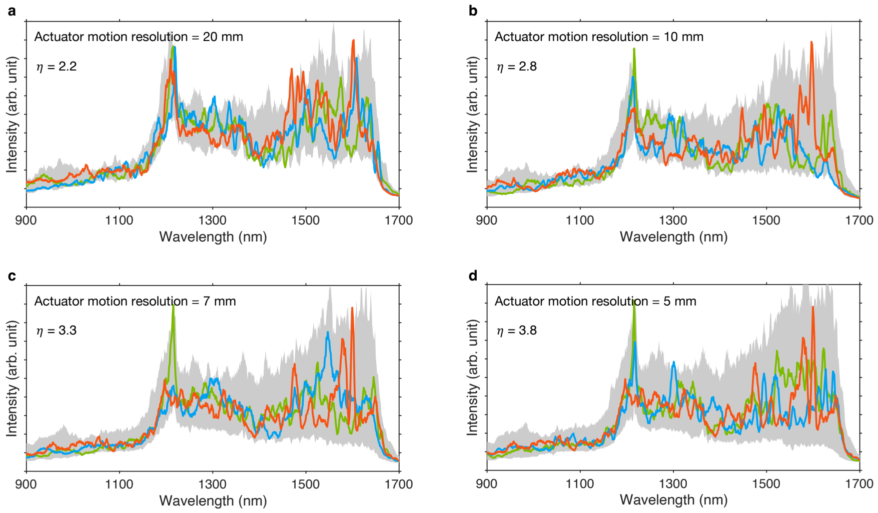

Supplementary Figure 6: **Spectral tunability of the fiber shaper with different actuator motion resolutions. a–d**, The output spectra range of the fiber-shaper-controlled SI MMF with the actuator motion resolution set at 20 mm (**a**), 10 mm (**b**), 7 mm (**c**), and 5 mm (**d**), respectively, all with 5 actuators and a displacement range of 20 mm. The input wavelength is set at 1300 nm. Source data are provided as a Source Data file.

Supplementary Note 3. Derivation of the multiphoton generation efficiency using a fiber source

The derivation follows one of the foundational references of two-photon fluorescence (2PF) imaging [10]. The motivation of this derivation is to provide insights into how the multiphoton signal was optimized through fiber-shaper-assisted temporal and spectral shaping of the pulse.

Given the same sample, we consider how the properties of the excitation source would affect the n-photon generation efficiency, which is described by the time-averaged fluorescence photon fluxes $S_n = \langle S_n(t) \rangle$. Since we assume only the excitation source varies, we can express $S_n(t)$ solely in terms of the intensity of the excitation $I(\boldsymbol{r}, t)$, that

$$S_n(t) \propto \int_V \mathrm{d}V I^n(\boldsymbol{r}, t), \tag{S2}$$

where V is the illuminated volume. We then express $I(\boldsymbol{r}, t)$ as $I(\boldsymbol{r}, t) = I_0(t)F(\boldsymbol{r})$ to further separate the temporal and spatial properties of the source, where $I_0(t)$ is the temporal distribution of the excitation source intensity and $F(\boldsymbol{r})$ is the normalized spatial distribution. Substitute it back to equation (S2), we have

$$S_n \propto g^{(n)} \langle I_0(t) \rangle^n \int_V \mathrm{d}V F^n(\boldsymbol{r}), \tag{S3}$$

where $g^{(n)} = \langle I_0^n(t) \rangle / \langle I_0(t) \rangle^n$ is the n-th order temporal coherence. In this work, the repetition rate (1 MHz) is the same for both the laser source and the MMF source, we therefore can further express the temporal term using the pulse energy E_p, pulse duration τ_p, and $g_p^{(n)}$ that only depends on the pulse shape:

$$g^{(n)} \propto \frac{g_p^{(n)}}{\tau_p^{n-1}}, \quad \langle I_0(t) \rangle \propto E_p. \tag{S4}$$

Finally, using the normalized PSF of the source to represent $F(\boldsymbol{r})$, we arrive at

$$S_n \propto \frac{g_p^{(n)} E_p^n}{\tau_p^{n-1}} \int \mathrm{d}V \mathrm{PSF}^n(\boldsymbol{r}). \tag{S5}$$

Equation (S5) implies that the properties of a source that would affect the multiphoton generation efficiency are: the pulse energy E_p, the pulse duration τ_p, the PSF distribution $\mathrm{PSF}(\boldsymbol{r})$, and the factor $g_p^{(n)}$ related to the pulse shape. In the imaging section, we simplify the model by assuming different sources share the same $g_p^{(n)}$ and $\mathrm{PSF}(\boldsymbol{r})$. As a result, the fiber shaper optimization (i.e., finding the optimal actuator configuration $s \in \mathcal{S}$) can be formulated as

$$\max_{s \in \mathcal{S}} \frac{E_p^n(s; \lambda)}{\tau_p^{n-1}(s; \lambda)}, \tag{S6}$$

where $\mathcal{S}$ is the space of all configurations.

Although not accounted for in the formulation, we observed that PSF can also be more confined with fiber shaper optimization. More advanced optimization scheme will be useful to further improve the spectral-temporal-spatial customization of the fiber source to push the fiber shaper to its limit.

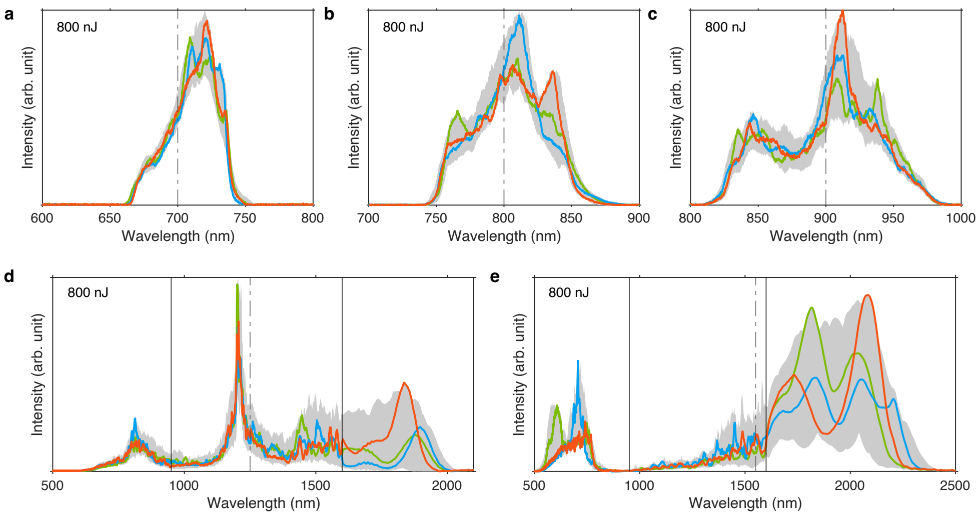

Supplementary Figure 7: **Fiber-shaper-controlled SI MMF with other input wavelengths. a–e**, Spectral broadening and tunability of varied input wavelengths of 700 nm (**a**), 800 nm (**b**), 900 nm (**c**), 1250 nm (**d**), and 1550 nm (**e**) in the same 30-cm-long fiber, with fixed input pulse energy of 800 nJ and the same set of macro-bending applied. Three representative spectra corresponding to three randomly chosen configurations are highlighted in distinct colors. Vertical solid lines mark the spectral range measured by different spectrometers. The dash-dotted line denotes the input wavelength. Source data are provided as a Source Data file.

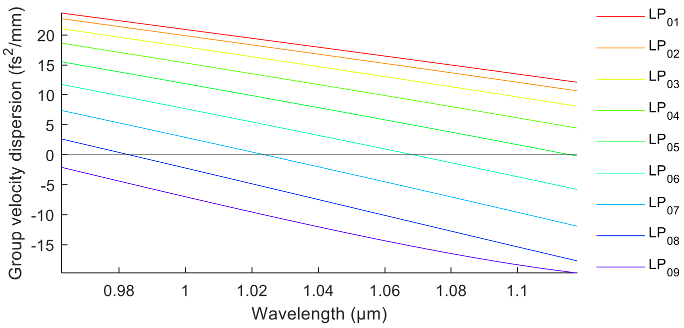

Supplementary Figure 8: Shifted zero-dispersion wavelengths of the higher-order modes.

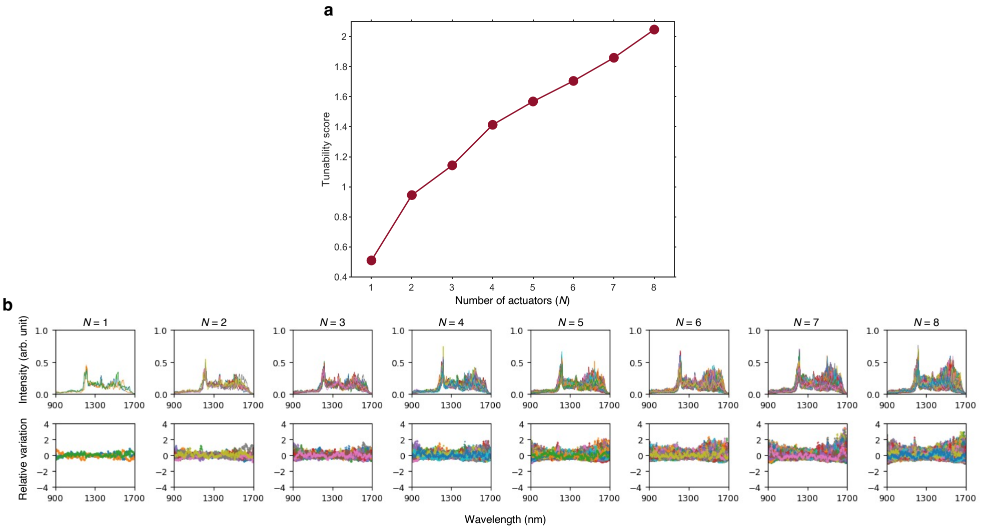

Supplementary Figure 9: **Spectral tunability with respect to the number of actuators. a**, The relationship between spectral tunability and the number of actuators. The tunability score is quantified by the mean spectral tuning range across the entire wavelength spectrum $\frac{1}{N}\sum_i \left[(I_{\lambda_i}^{\max} - I_{\lambda_i}^{\min})/\bar{I}_{\lambda_i}\right]$. **b**, All spectral data and the relative intensity variation at each wavelength $\left(I_{\lambda i} - \bar{I}_{\lambda_i}\right)/\bar{I}_{\lambda_i}$. Source data are provided as a Source Data file.

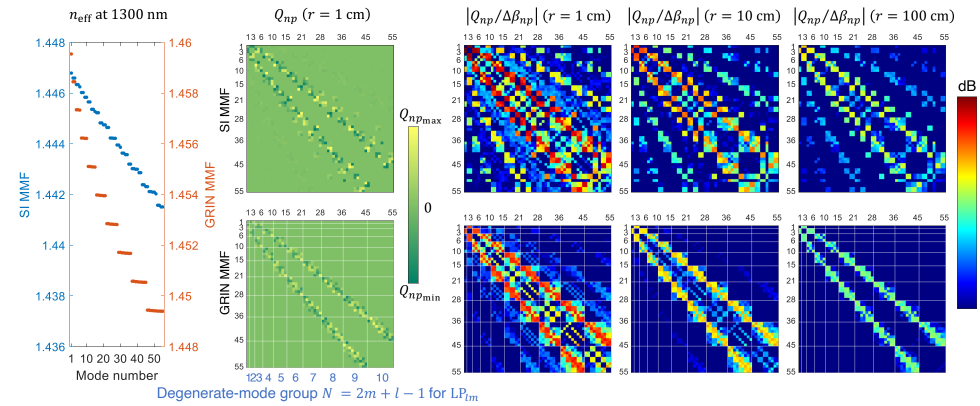

Supplementary Figure 10: **Simulation results of bending-induced mode coupling in step-index (SI) and gradient-index (GRIN) multimode fibers (MMFs).** The first two columns show the effective refractive index n_{eff} and the coupling coefficients Q_{np} for the first 55 spatial modes of the SI and GRIN MMFs.The right panel shows the linear mode coupling strength $\left|\frac{Q_{np}}{\Delta\beta_{np}}\right|$ [11] resulting from macrobending at various bending radii (1 cm, 10 cm, and 100 cm). Modes in the GRIN MMF are clustered as the degenerate-mode group, indicated by the solid white lines.

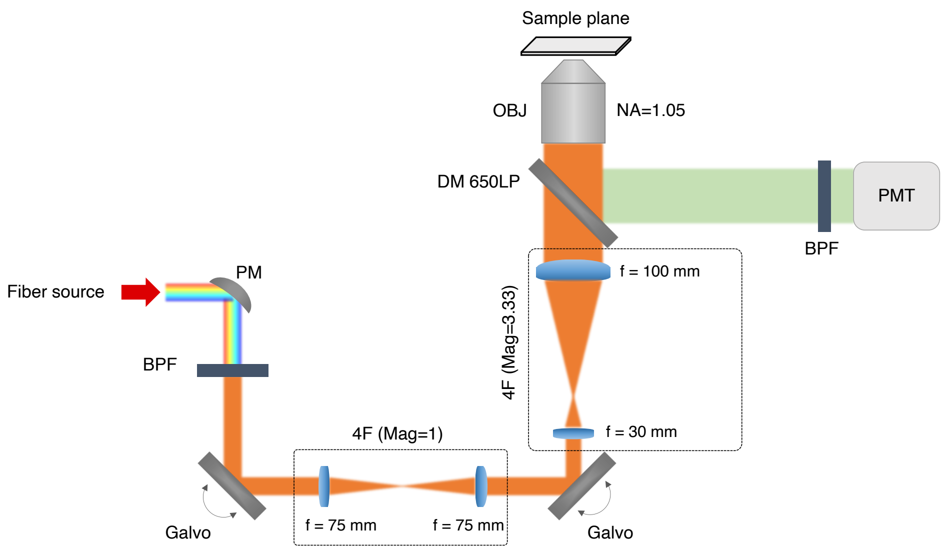

Supplementary Figure 11: **Schematic of the multiphoton imaging setup.** PM: parabolic mirror; BPF: bandpass filter; DM: dichroic mirror; LP: long pass; OBJ: objective lens; PMT: photomultiplier tube.

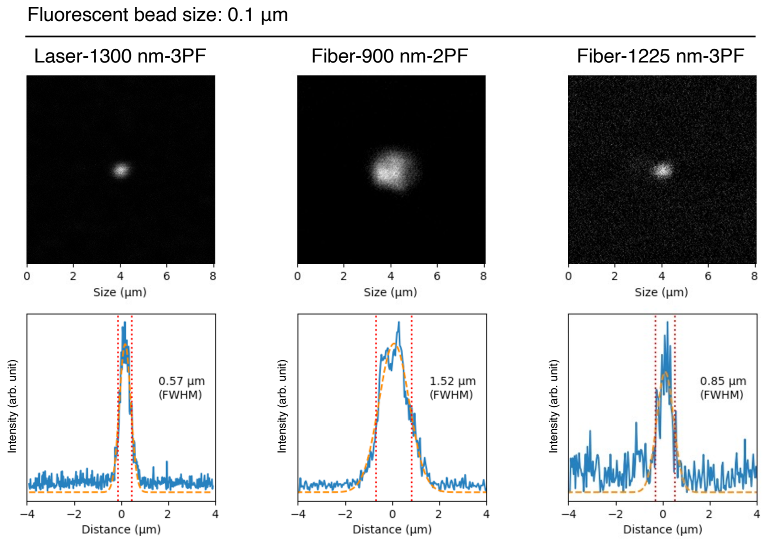

Supplementary Figure 12: **PSF measurement of the 3PF excitation with the laser (reference) and the 2PF and 3PF excitation with the fiber source.** The size of the bead is 0.1 µm.

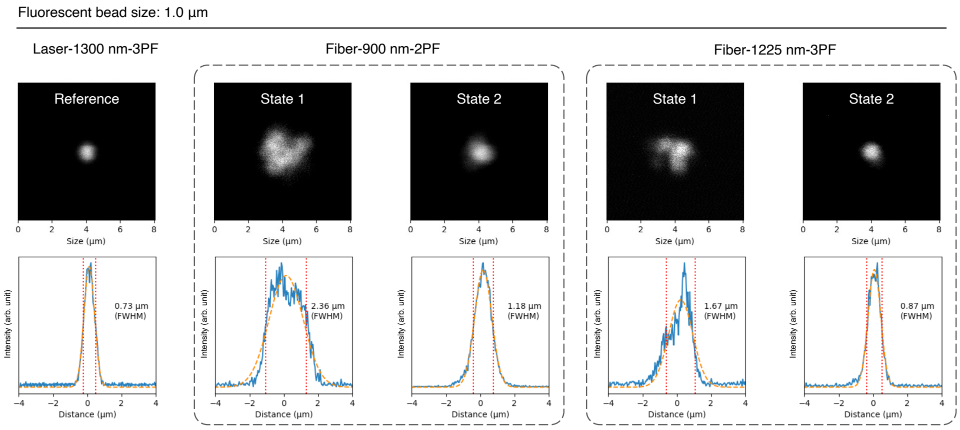

Supplementary Figure 13: **Qualitative PSF comparison of the 2PF excitation (900 nm) and the 3PF excitation (1300 nm) with the fiber source under random bending conditions induced by the fiber shaper's different states.** The size of the bead is 1 µm.

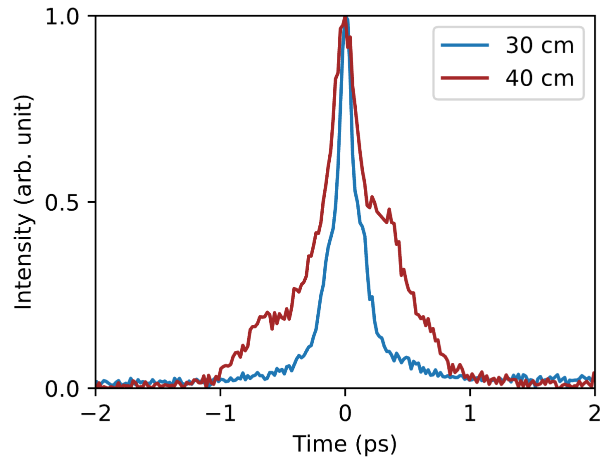

Supplementary Figure 14: **Representative results of the comparison of pulse durations out of straight step-index multimode fibers with two different fiber lengths.** The measurements were performed on the bandpass filtered (1100±25 nm) output with 1030 nm pump as an example.

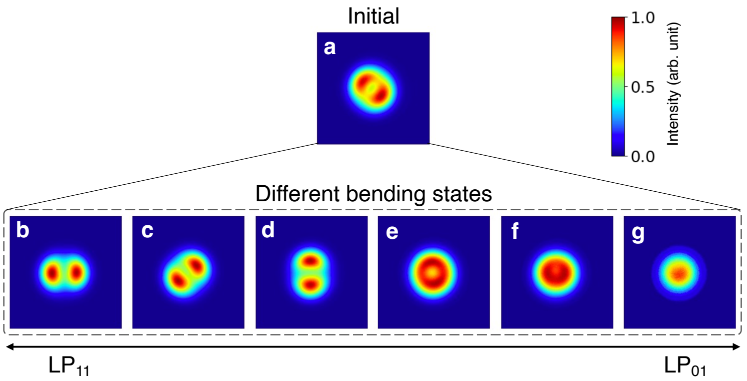

Supplementary Figure 15: **Normalized output spatial profiles of a 10-µm core step-index multimode fiber under various bending conditions. a**, Initial near-field intensity profile of the fiber output, showing a mixture of approximately three spatial linearly polarized (LP) modes. **b-g**, Near-field intensity profiles of the fiber output under various bending conditions, showing different combinations of the LP_{01} and the LP_{11} with two-fold spatial degeneracy. Each profile is individually normalized.

Actuator control codes

Actuator_control.py

```
###### Python key codes for actuator control ######
import tkinter as tk
from tkinter import filedialog, messagebox, ttk
import numpy as np
import time

from Motor.state_update import curr_state_update
from Motor.single_motion import *
from Spec.acquisition import spec_start, spec_stop


def auto_start(self):
    """Starts the automated process, handling initial setup
       and checks."""
    self.stop_flg = False
    if self.pause_flg:
        choice = messagebox.askquestion("Warning", "Start
           from initial state?")
        if choice == 'yes':
            self.pause_flg = False
        else:
            return

    # Calculate the state range based on maximum position and
       step size
    max_state = np.floor(self.max_pos / self.step)
    state_range = np.arange(-max_state, max_state + 1)

    method = self.auto_method.get()
    print(f"auto method = {method}")

    # Handle exhaustive search method
    if method == 'Exhaustive search':
        try:
            begin_state = [float(entry.get()) for entry in
               self.auto_begin_state_entry]
            end_state = [float(entry.get()) for entry in self
               .auto_end_state_entry]
        except ValueError:
            self.curr_state_lbl.config(text="Please enter
               valid auto states!", foreground="red")
            return
```

```
        # Validate the state ranges and sequence
        if any(state not in state_range for state in
            begin_state + end_state):
            self.curr_state_lbl.config(text="Warning: State
                out of range!", foreground="red")
            return

        if any(b > e for b, e in zip(begin_state, end_state))
             or (begin_state == end_state):
            self.curr_state_lbl.config(text="Warning: Invalid
                 start/end state sequence!", foreground="red")
            return

        # Generate list of states to visit
        begin_state = np.array(begin_state)
        end_state = np.array(end_state)
        states = [begin_state.copy()]
        next_state = begin_state.copy()
        while not np.array_equal(next_state, end_state):
            next_state[-1] += 1
            for i in range(self.n_motors-1):
                if next_state[-1-i] > end_state[-1-i]:
                    next_state[-1-i] = begin_state[-1-i]
                    next_state[-2-i] += 1
            states.append(next_state.copy())
        self.state_list = np.array(states).astype(int)

    # Handle list of states method
    elif method == 'List of states':
        state_list_text = self.auto_list_text.get('1.0', 'end
           -1c').rstrip().split('\n')
        try:
            state_list = [list(map(int, line.split(','))) for
                 line in state_list_text]
        except ValueError:
            self.curr_state_lbl.config(text="Invalid state
                list format!", foreground="red")
            return

        # Validate each state in the list
        for state in state_list:
            if any(num not in state_range for num in state)
                or len(state) != self.n_motors:
```

```
            self.curr_state_lbl.config(text=f"Invalid
                state {state} in the list!", foreground="
                red")
            return
    self.state_list = np.array(state_list)

# Set the beginning and end states
self.begin_state = self.state_list[0]
self.end_state = self.state_list[-1]

# Initialize variables for the automated process
self.state_idx = 0
self.start_time = time.time()
try:
    self.auto_dt = float(self.auto_dt_entry.get())
    self.auto_dt_lbl.config(foreground='black')
except ValueError:
    self.auto_dt_lbl.config(foreground='red')
    return

# Configure buttons and entry fields for the start state
auto_btn_status(self, 'Start')

# Move to the starting position
self.next_pos = self.begin_state * self.step
msg = move_to_state(self)
if msg:
    print(f"Moved to starting state {self.begin_state}\n
        ")
else:
    print("Error: Auto starting\n")

curr_state_update(self)

# Synchronize with the spectrometer if required
if self.gui.spec.spec_sync_var.get():
    error_flg = spec_start(self.gui.spec)
    if error_flg:
        auto_stop(self)
        return
# Begin the automated running process
self.gui.master.after(int(self.auto_dt*1000), lambda:
    auto_running(self))
```

```
def auto_stop(self):
    """Stops the automated process and resets the state."""
    self.stop_flg = True
    self.pause_flg = False
    self.state_idx = 0

    # Synchronize with the spectrometer if required
    if self.gui.spec.spec_sync_var.get():
        if not np.array_equal(self.motor_sync_state, self.gui
            .spec.spec_sync_state):
            self.gui.master.after(5, lambda: auto_stop(self))
            return
        else:
            spec_stop(self.gui.spec)

    # Configure buttons and entry fields for the stop state
    auto_btn_status(self, 'Stop')


def auto_pause(self):
    """Pauses the automated process."""
    self.pause_flg = True

    # Configure buttons and entry fields for the pause state
    auto_btn_status(self, 'Pause')


def auto_continue(self):
    """Continues the automated process from the paused state
        ."""
    self.pause_flg = False
    try:
        self.auto_dt = float(self.auto_dt_entry.get())
        self.auto_dt_lbl.config(foreground='black')
    except ValueError:
        self.auto_dt_lbl.config(foreground='red')
        return

    # Configure buttons and entry fields for the continue
        state
    auto_btn_status(self, 'Continue')

    self.gui.master.after(int(self.auto_dt*1000), lambda:
        auto_running(self))
```

```
def auto_running(self):
    """Main loop for running the automated process."""
    if self.stop_flg or self.pause_flg:
        return

    # Update the elapsed time display
    elapsed_time = time.time() - self.start_time
    minutes, seconds = divmod(int(elapsed_time), 60)
    self.auto_elapsed_t_lbl.config(text=f'Elapsed time: {
        minutes} min {seconds} s')

    # Synchronize with the spectrometer if required
    self.motor_sync_state = (self.curr_pos / self.step).
        astype(int)
    if self.gui.spec.spec_sync_var.get():
        if not np.array_equal(self.motor_sync_state, self.gui
            .spec.spec_sync_state):
            self.gui.master.after(5, lambda: auto_running(
                self))
            return

    # Process next state based on the selected method
    method = self.auto_method.get()
    self.state_idx += 1
    next_state = self.state_list[self.state_idx]

    # Move to the next position
    self.next_pos = next_state * self.step
    msg = move_to_state(self)
    if msg:
        print(f"Current state: {next_state}\n")
    else:
        print("Error: Auto running\n")
        return

    # Check if the current state is the end state and proceed
        accordingly
    if not np.array_equal(next_state, self.end_state):
        self.gui.master.after(int(self.auto_dt*1000), lambda:
            auto_running(self))
    else:
        self.gui.master.after(5, lambda: auto_end(self))
```

```
def auto_end(self):
    """Handles the end of the automated process."""
    # Synchronize with the spectrometer if required
    if self.gui.spec.spec_sync_var.get():
        if not np.array_equal(self.motor_sync_state, self.gui
            .spec.spec_sync_state):
            self.gui.master.after(5, lambda: auto_end(self))
            return
        else:
            spec_stop(self.gui.spec)

    # Loop or stop the process based on user choice
    if self.loop_var.get():
        self.gui.master.after(int(self.auto_dt*1000), lambda:
             auto_start(self))
    else:
        auto_stop(self)


def auto_btn_status(self, status):
    """Updates the status of the buttons based on the current
        state."""
    if status in ['Start', 'Continue']:
        # Logic for when the process is starting or
            continuing
        self.auto_start_btn.config(state='disabled')
        self.auto_continue_btn.config(state='disabled')
        self.auto_pause_btn.config(state='normal')
        self.auto_stop_btn.config(state='normal')
        [entry.config(state='readonly') for entry in self.
            auto_begin_state_entry + self.auto_end_state_entry
            ]
        self.auto_dt_entry.config(state='readonly')
    elif status == 'Stop':
        # Logic for when the process is stopping
        self.auto_start_btn.config(state='normal')
        self.auto_continue_btn.config(state='disabled')
        self.auto_pause_btn.config(state='disabled')
        self.auto_stop_btn.config(state='disabled')
        [entry.config(state='normal') for entry in self.
            auto_begin_state_entry + self.auto_end_state_entry
            ]
        self.auto_dt_entry.config(state='normal')
    elif status == 'Pause':
        # Logic for when the process is paused
```

```
    self.auto_start_btn.config(state='normal')
    self.auto_continue_btn.config(state='normal')
    self.auto_pause_btn.config(state='normal')
    self.auto_stop_btn.config(state='normal')
    [entry.config(state='readonly') for entry in self.
       auto_begin_state_entry + self.auto_end_state_entry
       ]
    self.auto_dt_entry.config(state='normal')
else:
    print('Not changing auto button status')
```

Actuator_running.ino

```
////// Arduino codes for actuator running //////
// Enable pins, HIGH for disable, LOW for enable

const int EnPins[] = {24, 38, 62, 30, 56}; // Z, Q, Y, E, X
    enable pins
const int StepPins[] = {26, 54, 46, 36, 60}; // Step pins
const int DirPins[] = {28, 55, 48, 34, 61}; // Direction pins

const int LED = 13; // LED pin

int dt = 1000; // [us] time interval between each pulse, no
    less than 1000 (Max starting frequency is 1000 pps)

int steps[5];
char signs[5];

byte buffer[10];

void setup() {
  Serial.begin(115200);
  pinMode(LED, OUTPUT);

  for (int i = 0; i < 5; i++) {
    pinMode(EnPins[i], OUTPUT);
    pinMode(StepPins[i], OUTPUT);
    pinMode(DirPins[i], OUTPUT);
  }

  Initial_device();
  // digitalWrite(LED, HIGH);
  // delay(1000);
  // digitalWrite(LED, LOW);
}

void loop() {

  if (Serial.available() >= 10) {
    Serial.readBytes(buffer, 10);
    for (int i = 0; i < 5; i++) {
      steps[i] = (int16_t)((buffer[i * 2 + 1] << 8) | buffer[
          i * 2]);
      signs[i] = (steps[i] > 0) ? '+' : '-';
      steps[i] = abs(steps[i]);
```

```
    }

    Allocate_Direction();

    // Move stepper motors
    for (int i = 0; i < 5; i++) {
      Move_Stepper(i, steps[i]);
    }

    Serial.println("Done");
  }
}

void Initial_device() {
  for (int i = 0; i < 5; i++) {
    digitalWrite(EnPins[i], HIGH);
    digitalWrite(StepPins[i], LOW);
    digitalWrite(DirPins[i], LOW);
  }
}

void Allocate_Direction() {
  for (int i = 0; i < 5; i++) {
    digitalWrite(DirPins[i], (signs[i] == '-'));
  }
}

void Move_Stepper(int motorIndex, int steps) {
  digitalWrite(EnPins[motorIndex], LOW); // LOW to enable
  for (int x = 0; x < steps; x++) {
    digitalWrite(StepPins[motorIndex], HIGH);
    delayMicroseconds(dt);
    digitalWrite(StepPins[motorIndex], LOW);
    delayMicroseconds(dt);
  }
  digitalWrite(EnPins[motorIndex], HIGH); // HIGH to disable
     for reducing energy consumption
}
```

References

[1] Wright, L. G., Renninger, W. H., Christodoulides, D. N. & Wise, F. W. Spatiotemporal dynamics of multimode optical solitons. *Optics Express* **23**, 3492–3506 (2015).

[2] Eftekhar, M., Lopez-Aviles, H., Wise, F., Amezcua-Correa, R. & Christodoulides, D. General theory and observation of cherenkov radiation induced by multimode solitons. *Communications Physics* **4**, 137 (2021).

[3] Renninger, W. H. & Wise, F. W. Optical solitons in graded-index multimode fibres. *Nature communications* **4**, 1719 (2013).

[4] Zitelli, M. *et al.* Multimode solitons in step-index fibers. *Optics Express* **30**, 6300–6310 (2022).

[5] Wu, Y., Bender, N., Christodoulides, D. N. & Wise, F. W. Highly multimode solitons in step-index optical fiber. *APL Photonics* **8** (2023).

[6] Dubietis, A., Tamošauskas, G., Šuminas, R., Jukna, V. & Couairon, A. Ultrafast supercontinuum generation in bulk condensed media (invited review). *arXiv preprint arXiv:1706.04356* (2017).

[7] Gaeta, A. L. Catastrophic collapse of ultrashort pulses. *Physical Review Letters* **84**, 3582 (2000).

[8] Horak, P. & Poletti, F. Multimode nonlinear fibre optics: theory and applications. *Recent progress in optical fiber research* **3** (2012).

[9] Wright, L. G. *et al.* Multimode nonlinear fiber optics: massively parallel numerical solver, tutorial, and outlook. *IEEE Journal of Selected Topics in Quantum Electronics* **24**, 1–16 (2017).

[10] Xu, C. & Webb, W. W. Measurement of two-photon excitation cross sections of molecular fluorophores with data from 690 to 1050 nm. *JOSA B* **13**, 481–491 (1996).

[11] Ho, K.-P., Kahn, J. M., Kaminow, I., Li, T. & Willner, A. Mode coupling and its impact on spatially multiplexed systems. *Optical Fiber Telecommunications VI* **17**, 1386–1392 (2013).
